# The Editor’s review of articles published from August to December 2019 in the *Southern African Journal of HIV Medicine*

**DOI:** 10.4102/sajhivmed.v21i1.1120

**Published:** 2020-08-24

**Authors:** David C. Spencer

**Affiliations:** 1 Division of Infectious Diseases, Department of Medicine, Helen Joseph Hospital, University of the Witwatersrand, Johannesburg, South Africa

## August 2019

1. Woods J, Moorhouse M, Knight L. A descriptive analysis of the role of a WhatsApp clinical discussion group as a forum for continuing medical education in the Eastern Cape, South Africa. S Afr J HIV Med. 2019;20(1):a982. https://doi.org/10.4102/sajhivmed.v20i1.982

**Editor’s comment:** In the year following my internship (1976), I worked at Mseleni Hospital, then a small 120-bed hospital in a remote corner of KwaZulu-Natal (KZN) province, South Africa. For much of the time, I was the only doctor. One of the highlights was the Thursday night radio call-in to discuss cases with colleagues at Manguzi and Bethesda, similar rural hospitals in northern KZN. I was a rookie. Darryl Hackland and Pat Garde (Bethesda) and Cliff Allward (Manguzi) were my lifeline. Another was the periodic weekend fly-in of Durban-based University of KZN academics who would assist with surgery and walk through the wards with me.

This observational study of the role of a WhatsApp group gives that story of a 21st-century twist. The goal of the authors was to assess the *educational value* of a WhatsApp group of 166 experienced and inexperienced doctors in rural public hospitals and clinics in the Eastern Cape province of South Africa and to ask whether the WhatsApp discussion was helpful and whether *informed consent and privacy rules* were breached. All the patients had complicated human immunodeficiency virus/tuberculosis (HIV/TB) co-infection. The study was undertaken between January 2016 and July 2017. The WhatsApp groups were given a short questionnaire and asked to submit answers anonymously. Although 86% of respondents replied that the group had given them ‘improved confidence and ability in managing sick patients’, and 52% said they used the guidance they had received ‘all the time’, many in the WhatsApp group (*n* = 74/166; 45%) never actually posted a response. Moreover, whilst answers such as ‘I use the guidance to manage patients’, ‘I refer to previous WhatsApp cases’, ‘I gained new clinical insights’ and so on reached statistical significance, that is, suggesting that clinical confidence had been increased, high odds ratios and wide confidence intervals suggest important limitations (Table 3 in the article). Does posting patient data risk a breach of doctor–patient ethics? Eighty-nine per cent of respondents agreed with the fact that informed consent would be required before posting patient-related data; however, in reality only 52% did this. And what about those registered on the programme who never appeared to participate? Was this a valuable learning experience for them? Perhaps, an analysis of the differences between the 50% who did not post cases and the 3% who did so frequently might answer this question.

2. Mugusi SF, Mopei N, Minzi O. Adherence to combination antiretroviral therapy among orphaned children in Dar es Salaam, Tanzania. S Afr J HIV Med. 2019;20(1):a954. https://doi.org/10.4102/sajhivmed.v20i1.954

**Editor’s comment:** Adherence to antiretroviral therapy (ART) is examined in this cross-sectional study of 216 Tanzanian orphans aged 2–14 years. All the children were HIV-positive and had been on nevirapine (NVP)-based ART for a minimum of 6 months. The study was conducted from June to September 2015. Adherence was measured in three ways: a 3-day recall of pill-taking behaviour (caregiver questioned), the historical regularity/reliability of clinic attendance and the monitoring of NVP-blood levels on study entry. Viral load levels are not supplied. Likely not available. On recall, 79.6% of children had not missed any doses, and 82.9% gave a history of regular clinic attendance. Yet, therapeutic levels of NVP, namely ≥ 3 µg/mL, were detected in only 72.2% of patients. On multivariate analysis, higher NVP levels were protective of unreliable clinic attendance (unadjusted odds ratios [uOR] 0.45, 95% confidence interval [CI] 0.21–0.95, *p* = 0.04) and linked positively to higher CD4 levels, namely > 25% and counts >500 cells/mm³, *p* = 0.001. Orphans missing both parents were at greater risk of low NVP blood levels, namely, uOR 1.37, 95% CI 0.69–2.68. Sub-therapeutic NVP levels were less likely amongst those orphans who were aware of their HIV status, that is, had experienced full disclosure (uOR 0.65, 95% CI 0.34–1.24). The limitations of this study are important: the cross-sectional design, the absence of an age-matched ‘non-orphan’ comparator-arm, reliance on caregiver ‘self-reporting’ and the wider lack of applicability of blood NVP levels to adherence management in Africa. The fact that viral loads are still not routinely available everywhere in sub-Saharan Africa is an inescapable subtext to this study. Is therapeutic NVP monitoring needed in Africa? It added value to this study. However, a wider role will be limited by costs and accessibility.

3. Vujanovic M, Brkic-Jovanovic N, Ilic D, et al. Associations of visceral fat thickness and anthropometric measurements with non-alcoholic fatty liver development in male patients mono-infected with human immunodeficiency virus. S Afr J HIV Med. 2019;20(1):a968. https://doi.org/10.4102/sajhivmed.v20i1.986

**Editor’s comment**: In this article from Serbia, 88 HIV-positive men on antiretroviral therapy (ART) were enrolled in a study evaluating a link between visceral fat thickness (VFT) as measured with abdominal ultrasound and the routine anthropometric measurements of obesity, cardiovascular risk and non-alcoholic steatohepatitis (NASH). The study took place over 18 months between September 2016 and April 2018. The average age of the men was 39.9 ± 9.9 years and the following anthropometric measurements were taken: waist and hip circumference (WC, HC), waist–hip and waist–height ratios (W/HipR, W/HtR) and the body mass index (BMI). Hepatic steatosis was diagnosed on sonography. Those with steatosis were more likely to have elevated random blood glucose levels, raised BMI and raised WC, HC, W/HipR and W/HtR in addition to elevated VFT (*p* < 0.001). Age ≥ 38.5 years was associated with an increased risk of the condition; 90.6% of those aged > 38.5 years with a VFT > 31.98 mm had hepatic steatosis. The authors discuss these results in the context of low- and middle-income countries where access to reliable non-invasive tests for hepatic steatosis is limited. The study limitations include its cross-sectional design, the absence of women and children and the lack of detailed information on antiretrovirals used by the men and the duration of their treatment.

4. Diana NE, Feldman C. Measles in adults: A comparison of hospitalised HIV-infected and HIV-uninfected patients. S Afr J HIV Med. 2019;20(1):a877. https://doi.org/10/4102/sajhivmed.v20i1.877

**Editor’s comment:** South Africa experienced an unusually large outbreak of measles between 2009 and 2011. In this descriptive study from the wards of the Charlotte Maxeke Johannesburg Academic Hospital, the authors present data on HIV-positive adults with laboratory-confirmed measles. Thirty-three adults with measles were identified, of whom 24 underwent HIV testing. Of the 24 tested for HIV, 18 (75%) were HIV-positive and six were HIV-negative. The remainder of the adult measles group (*n* = 9) were not tested. Most of the HIV-positive were women (13/18; 72%). Although the authors remarked that demographics, clinical findings and laboratory data between the HIV-positive and HIV-negative patients were similar, serious disease, for example, pneumonia and respiratory failure, was more frequent in the HIV-positive (OR 5.0, 95% CI 0.48–51.8, *p* = 0.34). The duration of hospital stay for the HIV-positive patients was significantly longer (*p* = 0.03), and of the three adult measles deaths, *all* were in the HIV-positive (OR 2.9, 95% CI 0.13–65.3, *p* = 0.56). The median CD4 count of the HIV-positive patients was 109 cells/mm³. Unfortunately, the authors do not provide further analysis, for example, individual CD4s, viral loads, antiretroviral therapy used and microbiology of the secondary infections. Do HIV-positive adults exposed to measles require re-vaccination or vaccination if this was missed in childhood? This is not addressed in this article, which is an important question. According to Loevinsohn (2019:836–844, in suggested reading below), ‘the measles vaccine should be given to potentially susceptible but asymptomatic HIV-positive adults and be considered for those with symptomatic HIV infection even if NOT severely immunosuppressed’.

## Suggested additional reading

- Loevinsohn G, et al. Measles seroprevalence and vaccine responses in human immunodeficiency virus-infected adolescents and adults: A systematic review. Clin Infect Dis. 2019 Aug 16;69(5):836–844. https://doi.org/10.1093/cid/ciy980.
- Moss WJ. Measles. Seminar. Lancet. 2017;390:2490–2502. https://doi.org/10.1016/S0140-6736(17)31463-0
- Measles vaccination: A WHO position paper. April 2017. Recommendations. Vaccine. 2019 Jan 7;37(2):219–222. https://doi.org/10.1016/j.vaccine20178.07.066).

5. Van Elsland SL, Peters RPH, Grobbelaar C, et al. Disclosure of human immunodeficiency virus status to children in South Africa: A comprehensive analysis. S Afr J HIV Med. 2019;20(1):a884. https://doi.org/10.4102/sajhivmed.v20i1.884

**Editor’s comment: Recommended reading.** In this cross-sectional study from the Western Cape, the authors ask the following questions: how many children know their HIV status and what factors assist our understanding of non-disclosure? It is a well-written report with data that deserve a wide audience. The total cohort was 185. All were on antiretroviral therapy and their ages ranged from 3 to 14 years. Most (145; 76.3%) had not experienced full disclosure, whilst 17 (8.9%) had experienced. A further 28 (14.7%) received ‘partial’ disclosure. The cross-sectional nature of the study, the small number of ‘disclosed’ children and the dependence on questionnaires, clinic records and caregiver’s reports would have introduced limitations but the take-home messages are worth noting: disclosure was more likely to have occurred amongst older children and those whose caregivers were more highly educated. The latter were more likely to be men, although less than 10% of the study’s caregivers were men. Indeed, disclosure was less likely if the caregiver was a woman, if children had detectable viral loads and if the child was still taking ‘syrup’ formulations of the antiretrovirals , namely, a younger group, if the child was noted by the caregiver to be non-adherent and if the child was on protease inhibitors, stavudine (d4T) and/or didanosine (ddI). This article provides the readers with credible information. Figure 1 in this article will also give HIV educators a useful outline to the important associations that promote disclosure/non-disclosure.

6. Schutz C, Ward A, Burton R, et al. False rifampicin results using Xpert MTB/RIF on urine samples in hospitalised HIV-infected patients. S Afr J HIV Med. 2019;20(1):a978. https://doi.org/10.4102/sajhivmed.v20i1.978

**Editor’s comment: Recommended reading.** This study is from colleagues in Cape Town. Urine samples were collected prospectively from HIV-positive patients with microbiologically proven active TB in two independent cohorts between 2012 and 2016. Multiple samples from each patient – including sputum, blood, tissue and urine – were subjected to culture, gene Xpert (including Xpert Ultra) and line probe analysis (LPA). A total of 1704 urine Xpert results were available from 1171 patients. Four hundred and sixteen (24.4%; 95% CI 22.4–26.5) of the urine Xpert results were positive for Mycobacterium tuberculosis (MTB) and 43/413 (10.4%) were rifampicin resistant on Xpert analysis. Of the latter group, 30/41 were confirmed to be truly rifampicin resistant, yielding a positive predictive value of 73.2% (95% CI 57.1–85/8). Urine tests from patients NOT on TB therapy at the time of assessment gave more true-positive rifampicin-resistant Xpert results (85.7%, 95% CI 67.3–96.0) than the urine of those on TB treatment (53.8%, 95 CI 25.1–80.8). About 11/43 urine results were falsely positive for rifampicin resistance (25.6%, 95% CI 13.5–41.2). Three patients in the urine Xpert rifampicin-resistant group were found to have concurrent rifampicin*-*sensitive TB on alternative specimens tested simultaneously, that is, ‘hetero-resistant TB’. This article is a stimulating read and feeds the reader’s mind – a compulsory reading for clinicians, particularly Infectious Diseases colleagues, fellows and registrars!!

7. Ekermans P, De Gama R, Kock C, et al. An unusual case of abdominal mycobacterial infection: Case report and literature review. S Afr J HIV Med. 2019;20(1):a993. https://doi.org/10.4102/sajhivmed.v20i1.993

**Editor’s comment: Highly recommended.** In this case report, Dr Ekermans and colleagues describe an HIV-positive 8-year-old’s experience of acquired immunodeficiency syndrome in South Africa. The author does a great job of describing the difficulties in confirming the diagnosis and isolating the organism. The histological and radiographic plates are superb: clear and compelling. The author tells this story with compassion and respect for his subject. The art and science of medicine shine on these pages. This is how we learn medicine, and how we become better doctors. I loved this read and recommend it to all who read this journal.

8. Atuhaire C, Taseera K, Spoor C, Cumber RY, Cumber SN. Knowledge and perceptions of male immigrants in Leeds (UK) towards male circumcision as an HIV-prevention strategy. S Afr J HIV Med. 2019;20(1):a823. https://doi.org/10.4102/sajhivmed.v20i1.823

**Editor’s comment**: Whilst the estimated prevalence of HIV infection in the United Kingdom (UK) is low, namely, ≤ 1.5 per 1000 persons, that of UK immigrants from Eastern and Southern Africa is far higher, namely, 25–50 per 1000 persons. Would medical male circumcision (MMC) be considered by these immigrants as a means of preventing HIV transmission? Only 10 persons were interviewed in a snowball recruitment study of contacts from a local church in Leeds, UK. The participants expressed little or no knowledge of circumcision as an HIV-preventive tool. Instead and despite the group’s roots from the epicentre of the epidemic, circumcision is still merely a ‘rite of passage’. Whilst the study limitations are obvious, it begs the question of the universality of HIV dogma. In the West, ‘Treatment as Prevention’ has replaced MMC. With current dogma promoting universal ‘test and treat (UTT)’ and ‘immediate ART for all’, should we be talking about MCC in high-income countries? And what of its future in middle- and low-income regions in the face of more effective preventive measures? And how effectively has MMC changed attitudes to HIV prevention in eastern and southern Africa?

### September 2019

9. Manickchund N, Du Plessis C, John M-A, et al. Case report. Emtricitabine-induced pure red cell aplasia. S Afr J HIV Med. 2019;20(1):a983. https://doi.4102/sajhivmed.v20i1.983

**Editor’s comment:** In this article, the authors report a female patient with pure red-cell aplasia. She was 35 years of age in 2014, pregnant, anaemic at baseline (haemoglobin [Hb] = 8.2 g/dL) and had a low CD4 count (83 cells/mm^3^). Two months after starting first-line ART, namely, tenofovir + emtricitabine (FTC) + efavirenz, she was found to be severely anaemic: Hb = 2.2 g/dL, normocytic normochromic. Her HIV infection was under control. Workup included a bone marrow examination, which revealed a pure red cell aplasia and a positive parvovirus B-19 polymerase chain reaction (PCR). The patient received intravenous immune globulin (IVIG) for 5 days and packed red cells. Over the next 11 months, she required multiple transfusions and six more courses of IVIG. Her ART was changed and the FTC stopped: tenofovir + abacavir + efavirenz, after which the anaemia resolved. However, the patient’s parvovirus B19-PCR remained positive (2017). A role for lamivudine (3TC) and emtricitabine in pure red cell aplasia has been suggested by multiple reports over the past two decades. This report is a reminder of this rare drug-related toxicity.

## Suggested additional reading

- Tsukamoto T. Hematopoietic stem/progenitor cells and the pathogenesis of HIV/AIDS. Front Cell Infect Microbiol. 2020 Feb 21;10:60. https://doi.org/10.3389/fcimb.2020.00060
- Durandt C, Potgieter JC, Mellet J, et al. HIV and haematopoiesis. S Afr Med J. 2019 Sep 10;109(8b):40–45. https://doi.org/10.7196/SAMJ.2019.vi09:8b.13829
- Knuesel SJ, Sawalla Guseh J II, Karp Leaf R, Ciaranello AL, Eng GM. Case 6-2018: A 35-year-old woman with headache, subjective fever, and anemia. N Engl J Med. 2018;378:753–760. https://doi.org/10.1056/NEJMcpc1712223

10. Cloete CM, Hampton J, Chetty T, et al. Evaluation of a health system intervention to improve virological management in an antiretroviral programme at a municipal clinic in central Durban. S Afr J HIV Med. 2019;20(1):a985. https://doi.org/10.4102/sajhivmed.v20i1.985

**Editor’s comment**: ‘What are the gaps in service delivery that allow for clinical failure/poor viral control?’ This is a detailed, prospective clinic-based study undertaken between 2011 and 2015. The investigators divided the study into three periods: pre-intervention, intervention and post-intervention. The intervention required checking every 10th patient file (*n* = 1538) with (1) an in-depth file review, (2) recording of viral loads (VL) and the ‘retention-in-care’ status of the client and (3) an assessment of the ‘viral load-management process’. Gaps were identified and interventions were implemented. Outcome measurements improved over the 4 years of the study, namely, the number of appropriate VL tests and the filing of results increased from 78% to 92% (*p* = 0.0009), the number of patients who accessed their VL result increased from 59% to 86% (*p* < 0.0001) and fewer patients, from 81% to 27%, required changes to antiretroviral therapy (ART) following the intervention. The detailed description in this report suggests a huge commitment from the clinic staff and the research team. Sustainable? The study required outside funding and the salaries of additional staff. Sustainable? Gaps are noted: continuing high patient volumes, the ongoing and urgent priority of ART-initiation, the need for and absence of dedicated pharmacists in HIV clinics and so on. The authors point out that the third UNAIDS 90 or (95)% goal is achievable, that is, reliable long-term VL suppression by 2030. This sounds optimistic. ‘Is this effort reproducible on a large scale?’

11. Kummerow M, Shaddock EJ, Klipstein-Grobusch K, et al. Unexpected low frequency of respiratory symptoms in an HIV-positive urban sub-Saharan population compared to an HIV-negative control group. S Afr J HIV Med. 2019;20(1):a1010. https://doi.org/10.4102/sajhivmed.v20i1.1010

**Editor’s comment:** This is a cross-sectional study of 547 adults living in Johannesburg between July 2016 and November 2017. Two-thirds of the patients were people living with HIV (PLWH) (*n* = 347, 63%). The remainder were HIV-negative matched controls. The median age of the patients was 37 years. Two-thirds (62%) were women. Recruits were asked about cough, cough accompanied by mucus or phlegm, breathlessness, wheeze and so on. The participants were also examined, had their blood tested (HIV status confirmed, HIV viral load [VL] and CD4 cell count) and completed a ‘quality of life’ questionnaire. The PLWH were subdivided into three groups: ‘the antiretroviral therapy (ART)-naïve’ (26%), ‘on first-line ART’ (24%) and ‘on second-line ART’ (50%). Those on ART were recruits from randomised controlled trials (RCTs) and demonstrated a high level (> 90%) of viral suppression. Their CD4 levels (median) were largely normal: namely, first-line ART, CD4 = 413.5 (range: 278.5–574.3) and second-line ART, CD4 = 619 (range: 429–798) cells/mm^3^, respectively. Those ‘initiating’ therapy, that is, naïve to ART when tested, also demonstrated relatively preserved median CD4 levels, namely, CD4 = 281 (range: 191–400.8) cells/mm^3^. Moreover, indeed, the authors note that ‘the frequency of respiratory symptoms did not differ by HIV status after adjustment for age and sex’. ‘Breathlessness (however) was associated with older age, female sex, obesity, a previous history of respiratory infection and airway hyper-reactivity (asthma)’. Chronic lung disease has been described in Africans living with HIV. The participants in this study were young and most had accessed reliable ART for several years, exhibited immune (peripheral CD4 cell) reconstitution and reliable viral suppression. How should HIV clinicians be monitoring the health of our patients’ respiratory tract? Will the Coronavirus disease 2019 (COVID-19) pandemic require a rethink of our patients’ respiratory safety?

## Suggested additional reading

- Desai SR, Nair A, Rylance J, et al. Human immunodeficiency virus-associated chronic lung disease in children and adolescents in chest radiographic and high-resolution computed tomographic findings. Clin Infect Dis. 2018;66(2):274–281. https://doi.org/10.1093/vid/cix778
- Shaddock EJ, Richards GA, Murray J. Lung fibrosis in deceased HIV-infected patients with pneumocystis pneumonia. S Afr J HIV Med. 2012;13(2):64–67.

12. Bharuthram N, Feldman C. The diagnostic utility of bone marrow examination in an infectious diseases ward. S Afr J HIV Med. 2019;20(1):a974. https://doi/org/10.4102/sajhivmed.v20i1.974

**Editor’s comment:** This is a retrospective review of consecutive bone marrow aspirate and trephine (BMAT) examinations of 327 patients admitted to the infectious diseases ward of a large academic hospital in Johannesburg from 2012 to 2014. Most of the patients (314;96%) were people living with HIV (PLWH). ‘What is the utility of BMAT in the context of HIV infection?’ A peripheral white blood cell (WBC) cytopenia of ≤ 4 × 10^9^/L predicted a ‘*unique*’ diagnosis (odds ratio [OR] 2.38, 95% CI 1.37–4.14, *p* = 0.002) and the likelihood of a *mycobacterial* infection (OR 2.11, 95% CI 1.28–4.41, *p* = 0.005). ‘Unique’ diagnoses mean diagnoses found only on BMAT despite extensive alternate investigation or achieved before the results of other tests were available or known. Unique diagnoses occurred in 77 (23.5%) patients and were *Mycobacterium tuberculosis* (MTB) in 17/77 (22%) and *Mycobacterium avium* complex (MAC) in another three patients. Three BMATs each provided ≥ 1X ‘unique’ diagnosis, for example, TB and cancer. Proven or suspected mycobacterial disease accounted for 57 BMATs with granulomas, culture-proven *MTB* without supportive histology in 50 and MTB confirmed with granulomas in 32 patients. The limitations of this study include its retrospective format, inherent case selection bias and, sadly, the absence of newer diagnostic tools such as sputum and urine MTB-rif-resistance gene-Xpert, gene-XPert Ultra and urine lipoarabinomannan (LAM). The latter is particularly disappointing as these molecular diagnostics are currently changing the face of clinical medicine.

13. Mehta UC, Van Schalkwyk C, Naidoo P, et al. Birth outcomes following antiretroviral exposure during pregnancy: Initial results from a pregnancy exposure registry in South Africa. S Afr J HIV Med. 2019;20(1):a971. https://doi/org/10.4102/sajhivmed.v20i1.971

**Editor’s comment: Recommended reading.** Although international first-line ART guidelines have replaced nevirapine (NVP) and efavirenz (EFV) with dolutegravir (DTG), concerns remain regarding the safety of ART in pregnancy. Dolutegravir is teratogenic in the first trimester of pregnancy. Women living with HIV and planning a family and those diagnosed with HIV in the first trimester should not use DTG. This article addresses the safety of NVP and EFV in pregnancy in a cohort of pregnant South African (SA) women.

In 2013, the SA National Department of Health promoted the introduction of a birth-outcomes registry amongst pregnant women and their infants exposed to ARVs. The authors report on the first 12 months of this programme (2013–2014). Two outcomes were assessed:

1. **Major congenital malformations (CMs)** following ARV exposure *in the first trimester* of pregnancy.
2. **Adverse birth outcomes (ABOs)**, namely, foetal death, preterm delivery, low birth weight, small for gestational age and neonatal death, following ARV exposure *at any time* during pregnancy.

Data were collected at the Prince Mshiyeni Memorial Hospital in Umlazi, Durban, South Africa. A total of 10 417 pregnancies and 10 517 birth outcomes were captured. The overall prevalence of HIV infection was 4013/10 417 (38.5%). A higher prevalence was noted in women > 35 years (640/1100; 58%) and in multigravida versus primigravid women (49.2% vs. 21.9%), respectively. The numbers of major CMs were small. About one-third of cases were in infants of mothers who were on ART (11/27; 29.7%). Compared to HIV-negative pregnant women unexposed to ARVs, first-trimester exposure to efavirenz in HIV-positive women did not increase the risk of CM (risk ratio [RR] 0.87, 95% CI 0.12–6.4, *p* = 0.895). However, first-trimester NVP exposure may increase the risk: RR 9.2, 95% CI 2.27–37.94, *p* = 0.002. This finding may have been influenced by confounders (e.g. small numbers) and thus requires more data or confirmation. The risk of ABOs was greater in infants of mothers with exposure to ART at any time throughout pregnancy versus HIV-uninfected mothers (RR 1.23, 95% CI 1.14–1.31, *p* < 0.001) but particularly where EFV or NVP use had started before the pregnancy. This report is published at a time when guidelines are changing. The non-nucleoside reverse transcriptase inhibitors (NNRTIs) are being phased out of first-line regimens. But women unable to take DTG are likely to be given EFV or perhaps NVP. This is a high-end paper that is informative and supports the long-term role of EFV in women for whom DTG is contraindicated.

## Suggested additional reading

- Zash R, Holmes L, Diseko M, et al. Neural tube defects and antiretroviral treatment regimens in Botswana. N Eng J Med. 2019 Aug;381(9):827–840. https://doi.org/10.1056/NEJMoa1905230
- The National Department of Health, The Republic of South Africa. 2019 antiretroviral treatment guidelines for the management of HIV in adults, pregnancy, adolescents, children, infants and neonates. October 2019. Dolutegravir Overview, ART Initiation, p. 8. Available from: https://www.health.gov.za

### October 2019

14. Cele MA, Archary M. Acceptability of short text messages to support treatment adherence among adolescents living with HIV in a rural and urban clinic in KwaZulu-Natal. S Afr J HIV Med. 2019;20(1):a976. https://doi.org/10.4102/sajhivmed.v20i1.976

**Editor’s comment:** This article reports the results of a small, questionnaire-based, cross-sectional, pilot study of 100 adolescents (aged 12–19 years) from two clinic sites – one urban (*n* = 50) and the other rural (*n* = 50) in KwaZulu-Natal, South Africa. Poor retention in care and unreliable treatment adherence challenge the success of antiretroviral therapy (ART) in this group of patients. Will text messaging remedy the problem? The authors confirm the widespread use (88%) of mobile devices amongst rural and urban respondents. Although two-thirds of participants were willing to receive their health information messages through mobile devices, others were unwilling or undecided. Higher education was found to be linked with greater mobile device usage. But who are those – people living with HIV – who are unwilling or undecided? Did the potential breach of privacy and the risk of unsanctioned disclosure via their smartphone inform the negative response? Forty-eight per cent of the cohort had never sent health-related short message services (SMSs) to or received (fewer) such messages from their clinic or health professionals. Who are these 12–19-year-olds who are unwilling or undecided? Are these the ones who will be lost to care and fail adherence? How do we ensure these also become confident, understand their rights and are assisted to adhere to ART?

15. Van Wyk B, Davids L-A. Challenges to HIV treatment adherence among adolescents in a low socio-economic setting in Cape Town. S Afr J HIV Med. 2019;20(1):a1002. https://doi.org/10.4102/sajhivmed.v20i1.1002

**Editor’s comment:** This is a descriptive record of challenges faced by 15 adolescents (aged 10–19 years) living with HIV since birth, and receiving support from a primary care clinic in the greater Cape Town district. The participants were interviewed in 2016 and had been on antiretroviral therapy for a minimum of 6 months. Group and individual discussion focused on barriers to and facilitators of adherence. The themes identified by the authors are not new: the conflict between school and clinic, the need for greater ‘HIV-competency’ of households and the provision of adolescent-friendly health services. Limitations are acknowledged: small numbers, incomplete data saturation and the absence of a wide spectrum of views including that of defaulters. However for me, the strength of this study includes the verbatim comments of the participants. For a brief moment, the reader hears what it is like to be young and stigmatised and shamed by HIV and AIDS. This is why adherence is so difficult. It is not simply a matter of healing our youth; it is rather about society and the ongoing wider response to people living with HIV.

16. Moodley K, Bill PLA, Patel VB. Motor lumbosacral radiculopathy in HIV-infected patients. S Afr J HIV Med. 2019;10(1):a992. https://doi.org/10.4102/sajhivmed.v20i1.992

**Editor’s comment:** This is a short report of 11 young (median age = 29 years) people living with HIV naïve to antiretroviral therapy (ART), who experienced a slowly progressive, bilateral and symmetrical, isolated, lower motor neurone weakness of the lower limbs. The latter were areflexic and flaccid. The remainder of the neurological examination, including higher function, sensation and sphincter control, were normal. A diagnosis of subacute motor lumbosacral radiculopathy was made. The mean duration of symptoms was 6.5 months (interquartile range [IQR] 3–7.5 months). Six were female patients. Cerebrospinal fluid (CSF) was notable for an elevated protein and the presence of mononuclear cells. Tests for malignancy and various infecting organisms were negative. The group’s median CD4 cell count was 327 cells/mm^3^ (IQR 146 cells/mm^3^ – 457 cells/mm^3^). Unfortunately, serum and CSF HIV viral load levels were not drawn. On magnetic resonance imaging (MRI), gadolinium enhancement was visible in the lumbar ventral roots. Electromyography (EMG) confirmed abnormal activity of the lumbar and lower limb muscles. All the patients were treated for up to 4–6 weeks, with large amounts of oral prednisone (1.5 mg/kg/day). Steroids were sometimes given for longer periods. No steroid toxicity and no intercurrent (opportunistic) infection (e.g. TB and fungal) were reported. Antiretroviral therapy was not started immediately and no patient was on ARVs at the time of diagnosis. Ninety-one per cent of patients recovered within 3.4 months. All except one with mild residual weakness were ‘normal’ at the final 18-month follow-up visit. The authors – from the Neurology Department of the University of KwaZulu-Natal (UKZN), Durban, South Africa – discuss the differential diagnosis and point out that since the rollout of ‘Universal Test and Treat’ in 2017, few additional cases have been reported.

17. Laughton B, Naidoo S, Dobbels EFMT, et al. Neurodevelopment at 11 months after starting antiretroviral therapy within 3 weeks of life. S Afr J HIV Med. 2019;20(1):a1008. https://doi.org/10.4102/sajhivmed.v20i1.1008

**Editor’s comment. Recommended reading.** This is an important, prospective, observational study of 29 infants born to mothers living with HIV (MLWH). All infants were started on antiretroviral therapy (ART) within 21 days of their birth. Twenty-four of the mothers (83%) were on ART at the time of delivery. Twenty-three (79%) infants were females. Infant viral load (VL) level at birth was 3904 (The median infant VL level at birth was 3904 (range, 259-16,022) copies/mL. Viral suppression (VL < 400 copies/mL) on ART occurred at 19.1 weeks (median, range 15, 36) of age. The Global Griffiths Mental Development Scales (GMDS), an early neurodevelopmental assessment tool, found the infant’s developmental scores to be normal at 11.5 ± 0.8 months. This was despite the fact that 9/29 (31%) infants had a detectable bloodstream VL at the time. Of the five central nervous system (CNS) domains assessed, locomotor skills scored the lowest and hearing and language the highest. The authors acknowledge that this study is small and the results are preliminary. Nonetheless, these data suggest that ART started at this extremely young age is safe and beneficial. The authors inform us that a larger study is already underway. This article is another milestone along the way to beating the virus and to the well-being of future generations,

18. Archary M, Fairlee L, Slogrove A. Opinion piece. Current perspectives on paediatric HIV management from the Mexico International AIDS Society Conference, 2019. S Afr J HIV Med. 2019;20(1):a1027. https://doi.org/10.4102/sajhivmed.v20i1.1027

**Editor’s comment: Recommended reading.** This is a summary of presentations and discussions held at the following meetings: the 11th International Workshop on Pediatric HIV and the 5th Workshop on Children and Adolescents *HIV-Exposed and Uninfected*, and the International AIDS Society (IAS) Conference in Mexico, July 2019. The authors remark that despite general success in controlling vertical transmission, there were 160 000 new global paediatric HIV infections in 2018. They further add that ‘sub-Saharan Africa is struggling with meeting UNAIDS 90-90-90 goals for children and adolescents living with HIV’. This is nevertheless an optimistic report that focuses on antiretroviral treatment and the prevention of vertical transmission of HIV.

**New antiretrovirals and a new delivery system:** *GS-6207* is the first of the capsid-inhibitor ARV class but currently no paediatric studies have been reported. Adult trials are promising. Treatment = long-acting, s/c administration every 3 months. *MK-8591*, the first nucleoside reverse transcriptase ***TRANSLOCATION*-inhibitor.** No paediatric data but adult studies = prolonged intracellular half-life and low once a week dosing. No cross-resistance to other nucleoside-reverse transcritpase inhibitors (NRTIs). An adult fixed-dose combination (FDC) in trials: MK-8591+doravirine. A paediatric formulation is possible. Tenofovir alafenamide (TAF) in children, a new NRTI with lower renal and bone demineralisation risk and an FDC formulation for children aged > 6 years and weighing ≥ 25 Kg: TAF/FTC/elvitegravir+cobicistat. Of a variety of novel drug delivery systems, the adult transdermal adhesive micro-needle skin patches that deliver monthly cabotegravir offer new therapeutic or prevention options with potential for paediatric application.

**Antiretroviral drug efficacy and safety: Birth defects.** The risk of neural tube defects in infants exposed to dolutegravir (DTG) during periconception (first-trimester of pregnancy) appears to be confirmed with updated data from Botswana’s Tsepamo Birth Surveillance Study. Nevertheless, the World Health Organization recommends DTG-based antiretroviral therapy (ART) for all women of child-bearing potential who are living with HIV. Women who are not pregnant must be counselled regarding contraception. And counselling of all women MUST ensure that the decision for either a DTG or an efavirenz (EFV)-based ART is a fully INFORMED DECISION and decided ahead of the start of ART. Children who are HIV-exposed but uninfected (CHEU) enrolled in the International Antiretroviral Pregnancy Registry (APR) had lower birth weight- and length-for-age compared to unexposed, uninfected children. Zimbabwe’s SHINE study also reported stunting and mortality risk to CHEU (see comment summary 13 above).

**Metabolic effects of ART.** Exposure of adults to ART (the South African ADVANCE study) uncovered significant weight gain in all study arms but especially those given both DTG+TAF/FTC. What drug-related toxicity is emerging and what does this mean for children on these drugs?

**Public health, ART and children**. Paediatric retention in care and viral load (VL) suppression rates are suboptimal in southern Africa. Adolescent mortality and morbidity risk is too high. According to the International Epidemiologic Databases to Evaluate AIDS – Southern Africa (IeDEA-SA) collaboration data 2004–2017, ‘children lag behind’.

Readers are encouraged to check out this ‘Opinion Piece’ for themselves. Its background is the success of the prevention of mother-to-child transmission of HIV. Its reality is the day-to-day management of young people living with HIV.

19. Dunlop JL, Slemming W, Schnippel K, et al. Breast abnormalities in adolescents receiving antiretroviral therapy. S Afr J HIV Med. 2019;20(1):a1017. https://doi.org/10.4102/sajhivmed.v20i1.1017

**Editor’s comment:** This is a retrospective report of abnormal breast development in adolescents aged 10–19 years. All were on antiretroviral therapy (ART). The article reflects January–December 2014 clinical records of 631 Johannesburg-based adolescents living with HIV (ALWH). Of the patients, 37 (5.9%) had ‘abnormal’ breasts. Of those with abnormal breasts, most (24; 65%) were men (*p* = 0.043). Median duration of ART was 4.9 years. The older adolescents (*p* < 0.0005) and those on efavirenz (EFB)-based ART (*p* = 0.016) were more likely to have breast problems. Ninety-two per cent (34/37) were on EFV, whilst the remaining 8% were on lamivudine (3TC) monotherapy, that is, they were likely to have been on EFV previously. (The prevalence of EFV use amongst those with normal breasts – the comparator group – was lower, namely *n* = 384/594;74.3%). The use of boosted-protease inhibitors, such as lopinavir or ritonavir, atazanavir and darunavir, was not associated with breast changes. Although lipodystrophy was recorded in 46.3% and gynecomastia in 29% of those with breast changes, body mass index-confirmed overweight or obesity was found in only eight (19.5%) participants. More than 70% had viral suppression (VL < 50 copies/mL) and immune reconstitution (CD4 count ≥ 500 cells/mm^3^). Most of the 37 adolescents took their pills. The file review indicated that clinic physicians ‘corrected’ the problem by replacing EFV with nevirapine (NVP). This hardly solved the problem. The authors remark that few were referred for additional breast care. And of the three who were referred, none received any definitive intervention. This article has also limitations. Retrospective studies have a tendency to accumulate data gaps with the passage of time. Did the breast abnormalities ever resolve? The use of EFV is widespread in sub-Saharan Africa. Are these toxicities still being seen? Is anyone culpable? After all, these are iatrogenic adverse events.

20. Govender NP, Meintjes G, Mangena P, et al. Southern African HIV Clinicians Society guideline for the prevention, diagnosis and management of cryptococcal disease among HIV-infected persons: 2019 update. S Afr J HIV Med. 2019;20(1):a1030. https://doi.org/10.4102/sajhivmed.v20i1.1030

**Editor’s comments:** I recommend this guideline paper to all. It is a must-read and is up there with the best.

21. Spencer DC, Krause R, Rossouw T, et al. Palliative care guidelines for the management of HIV-infected people in South Africa. S Afr J HIV Med. 2019;20(1):a1013. https://doi.org/10.4102/sajhivmed.v20i1.1013

**Editor’s comment:** This is a guideline paper and I thank the colleagues who helped me with this one. If you are interested in this field of HIV medicine, please contact the HIV Clinicians Society. We are eager to start an interest group to develop the field further.

22. Kaplan S, Nteso KS, Ford N, Boulle A, Meintjes G. Loss to follow-up from antiretroviral therapy clinics: A systematic review and meta-analysis of published studies in South Africa from 2011 to 2015. S Afr J HIV Med. 2019;20(1):a984. https://doi.org/10.4102/sajhivmed.v20i1.984

**Editor’s comment: Recommended reading**. This review article provides a meta-analysis of public sector South African antiretroviral therapy (ART) studies that address loss to follow-up (LTFU) and mortality data. This is an important article and will inform the epidemiology and practice of ART in South Africa in the years ahead.

The data are derived from 48X adult, 15X paediatric and 4X pregnancy studies completed between January 2011 and October 2015. The study limitations are acknowledged: non-homogeneous data sources, namely, clinics, hospitals, rural and urban sites; and non-standardised definitions of LTFU across sources. When is a client lost to follow-up? After 3, 6 or 12 months since their last visit? The initiation of ART itself was in flux at the time: baseline CD4 counts varied and treatment varied between private and state providers. Were ‘silent transfers’ excluded from the LTFU data, that is, clients who move between clinics without informing staff and who are not truly LTFU?

The median cohort study size was 3737 persons. Median adult age and baseline CD4 count at ART initiation were 35.8 years and 121 cells/mm^3^, respectively. The median age of ART initiation in children was 4.2 years. The ‘defined’ follow-up time varied from 9 weeks to 5 years and the meta-analysis indicated no difference in LTFU estimates at 3-, 6- or 12-month census. The overall median mortality at 1 year was 7.9% (IQR 4.1–11.4; range 0% – 26%) and the median LTFU at 1 year was 12.8% (IQR 7.9% – 22.0%, range 0.2% – 43.1%). Aggregate meta-analysis LTFU estimates at 1 year were 11.6% (95% CI 11.4% – 11.7%) in the adult studies, 30.0% (95% CI 28.7% – 37.4%) for the pregnant cohorts and accounted for 7.5% (95% CI 6.7% – 8.2%) of the paediatric data. The 5-year LTFU estimate was 25% (95% CI 24.8% – 25.4%) based on three adult studies. In their concluding remarks, the authors indicate their support for the standardisation of the LTFU definition to 180 days (6 months).

Thank you for reading through this summary of articles published in the *Southern African Journal of HIV Medicine* August–December 2019. Similar summaries are available for the first half of 2019. If you have suggestions and improvements, please contact me at editor@sajhivmed.org.za.

**Dr David Spencer**

Editor-in-Chief

Southern African Journal of HIV Medicine

